# Viscoelastic Hydrogels Governed by Molecular Interactions and Mechanochemical Effects

**DOI:** 10.3390/polym18091126

**Published:** 2026-05-02

**Authors:** Wenjie Zhang, Dianrui Zhang, Haocheng Niu, Junsheng Zhang, Yiran Li

**Affiliations:** School of Chemistry and Chemical Engineering, Ningxia University, Yinchuan 750021, China

**Keywords:** viscoelastic hydrogels, multiscale mechanics, energy dissipation, single-molecule force spectroscopy, mechanochemistry

## Abstract

Hydrogels, particularly those based on polymer networks, exhibit complex mechanical behaviors arising from the interplay between network architecture, molecular interactions, and external stimuli. In particular, their viscoelasticity, energy dissipation, and nonlinear mechanical responses arise from the dynamic nature of crosslinking and multiscale relaxation processes. This review provides a comprehensive overview of hydrogel mechanics from a multiscale perspective, covering viscoelastic behavior, relaxation dynamics, energy dissipation mechanisms, nonlinear deformation, and fracture properties. We summarize recent advances in experimental characterization, including bulk rheology and single-molecule force spectroscopy, and discuss how molecular-level interactions, bond kinetics and mechanochemical processes contribute to macroscopic mechanical performance. In addition, theoretical models and constitutive frameworks describing transient and dynamic polymer networks are critically evaluated to bridge microscopic dynamics with bulk responses. Emerging strategies that integrate dynamic bonding and force-responsive elements are also discussed in the context of tailoring mechanical adaptability and functionality. Finally, we outline current challenges and future directions toward the rational design of hydrogels with tunable viscoelasticity, enhanced mechanical robustness, and programmable mechanical functions.

## 1. Introduction

Hydrogels are soft materials formed by three-dimensional networks of hydrophilic polymers that retain structural integrity while absorbing substantial amounts of water, often exceeding 90% of their weight. At the molecular level, the primary polymer chains are constructed through covalent bonds between monomer units, which define the permanent backbone of the network and ensure structural integrity. This unique combination of high-water content and solid-like mechanical behavior endows hydrogels with excellent biocompatibility and highly tunable mechanical properties, making them widely applicable in biomedicine, tissue engineering, flexible electronics, soft robotics, and adhesive materials [1]. In these applications, mechanical performance, particularly the time-dependent response under complex mechanical environments, plays a decisive role in determining functionality. Accordingly, understanding and controlling the mechanical behavior of hydrogels has long been a central research focus, with early studies primarily centered on equilibrium swelling, network structure, and static elastic moduli, often described using classical rubber elasticity theory.

However, beyond equilibrium elasticity, hydrogels inherently exhibit viscoelastic behavior, which arises from the dynamic nature of polymer networks and governs their time-dependent mechanical responses. In contrast to the covalent backbone, these behaviors are primarily regulated by reversible interactions, including physical crosslinking, supramolecular associations, and dynamic covalent bonds, which enable network rearrangement under deformation. Unlike purely elastic solids, hydrogels display coupled viscous and elastic characteristics, enabling both energy storage and dissipation [2]. As a result, viscoelasticity provides a fundamental framework linking molecular interactions, network dynamics, and macroscopic mechanical properties. It is therefore essential for understanding key mechanical phenomena such as stress relaxation, creep, hysteresis, and fatigue resistance, as well as for establishing structure-property relationships across multiple time and length scales.

The importance of viscoelasticity is further highlighted by its ubiquity in biological systems. Most native tissues, including brain [3,4], lung [5], skin [6], liver [7,8], muscle [9], cartilage [10,11], and tendon [12,13], exhibit pronounced viscoelastic behavior. Cells within these tissues respond to mechanical stimuli in a time-dependent manner, displaying stress relaxation, creep, and frequency-dependent deformation without compromising structural integrity [14]. Inspired by these biological characteristics, hydrogel research has progressively shifted from focusing on static mechanical properties toward understanding and regulating time- and rate-dependent responses under complex loading conditions [15,16,17]. In particular, increasing evidence demonstrates that the viscoelastic properties of the extracellular matrix play a decisive role in controlling cell behavior, including stem cell differentiation, cell migration, and tissue morphogenesis. Notably, stress relaxation has emerged as a key regulatory factor that can be decoupled from stiffness, enabling independent investigation of how the temporal dimension of mechanical cues influences biological processes.

Despite these advances, a fundamental challenge remains in establishing quantitative and predictive relationships between molecular-scale dynamics and macroscopic viscoelastic behavior. Specifically, how network architecture, crosslinking chemistry, and polymer chain dynamics collectively determine relaxation processes, energy dissipation, and nonlinear mechanical responses is still not fully understood. Addressing this challenge requires a multiscale framework in which molecular bond dynamics-particularly the lifetime and reversibility of crosslinking interactions-are quantitatively linked to network relaxation processes and bulk mechanical response.

**Figure 1:**
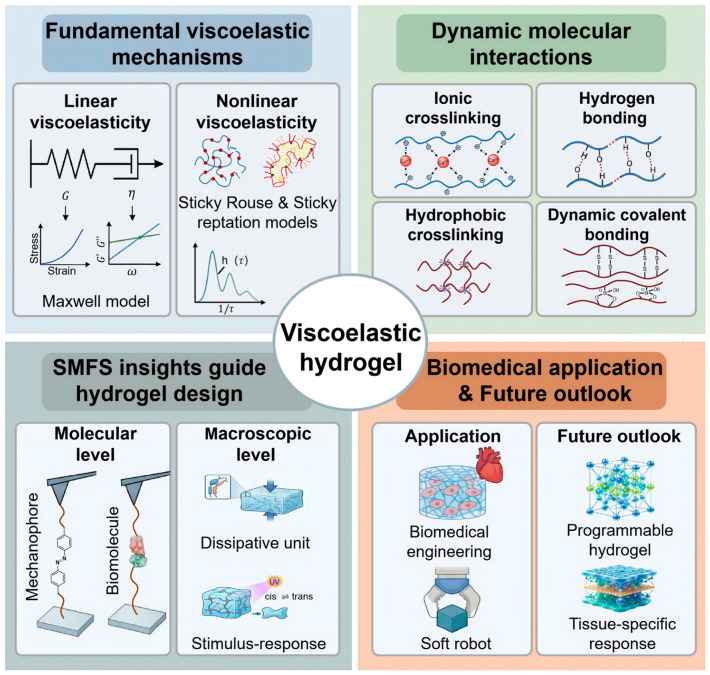
Schematic overview of viscoelastic hydrogels: from fundamental viscoelastic mechanisms, dynamic molecular interactions, and single-molecule force spectroscopy (SMFS)-guided design to biomedical applications and future outlook.

In this review, we provide a comprehensive overview of recent advances in hydrogel mechanics, with a particular emphasis on viscoelasticity as a central governing principle (Figure 1). We discuss the fundamental physical models and theoretical frameworks that describe viscoelastic behavior, followed by its macroscopic manifestations and key mechanical characteristics. We then summarize current strategies for tuning hydrogel viscoelasticity through dynamic interactions, including hydrogen bonding, physical crosslinking, and metal-ligand coordination. In addition, we highlight recent progress in bridging molecular-scale mechanics and mechanochemistry-regulated bulk mechanical performance, offering insights into the multiscale origins of hydrogel behavior. Finally, we outline emerging challenges and future opportunities in the design of next-generation functional hydrogels.

## 2. Fundamental Frameworks of Viscoelasticity in Hydrogel Networks

**Figure 2:**
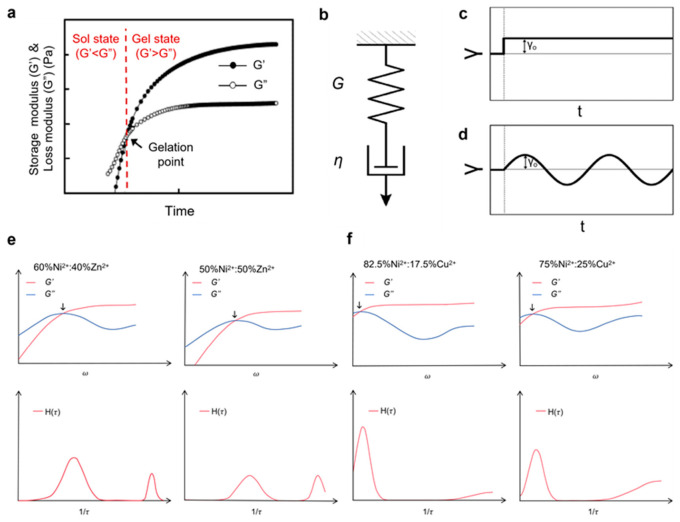
Rheological characterization and modeling of hydrogel viscoelasticity. (**a**) Rheological characterization of the sol–gel transition process of a hydrogel precursor (The black arrow indicates the intersection point of $G^{\prime }$ and $G^{\prime \prime }$). Copyright 2021, Springer Nature [26]. (**b**) Schematic representation of the Maxwell model, consisting of a linear elastic spring (modulus G) and a viscous dashpot (viscosity η) connected in series. Copyright 2023, Royal Society of Chemistry [27]. (**c**) Stress relaxation response of the Maxwell model under a step strain. Copyright 2023, Royal Society of Chemistry [27]. (**d**) Frequency-dependent linear viscoelastic response of the Maxwell model under oscillatory deformation ($G_{0}=1$). Copyright 2023, Royal Society of Chemistry [27]. (**e**,**f**) Different energy dissipation modes by tuning the active crosslinks. Two distinct relaxation modes in $G^{\prime \prime }(\omega )$ are fitted to H(τ) (bottom panels), confirming that a double log-normal spectrum accurately describes the data (solid/dashed lines, top panels) (The black arrow indicates the intersection point of $G^{\prime }$ and $G^{\prime \prime }$). These schematic figures were redrawn based on Grindy S C et al., 2015 [28].

Viscoelasticity describes the time-dependent mechanical response of materials arising from the interplay between elastic energy storage and viscous dissipation. In polymer networks such as hydrogels, this behavior originates from the dynamic nature of chain conformations, crosslinking interactions, and network rearrangements across multiple time scales. Under constant stress, viscoelastic materials exhibit creep, characterized by a gradual increase in strain, whereas under constant strain they undergo stress relaxation, reflecting the progressive redistribution of internal stresses (Figure 2a) [18]. These responses are fundamentally linked to the spectrum of relaxation processes within the material and can be quantified through time- and frequency-dependent mechanical functions.

The Maxwell model provides the simplest theoretical description of linear viscoelasticity [19], representing the material as a spring and dashpot connected in series. Its constitutive equation is given by (Figure 2b):

$$\sigma \left(t\right)+\frac{\eta }{G}\frac{d\sigma \left(t\right)}{dt}=\eta \frac{d\gamma \left(t\right)}{dt}$$

where G and η denote the elastic modulus and viscosity, respectively, and $\tau_{c}=\eta /G$ defines the characteristic relaxation time. For a step strain, the relaxation modulus follows a single-exponential decay (Figure 2c):

$$G\left(t\right)=G_{0}\exp\left(-\frac{t}{\tau_{c}}\right)$$

Under oscillatory shear, the complex modulus is expressed as (Figure 2d):

$$G^{*}\left(\omega \right)=G^{\prime }\left(\omega \right)+iG^{\prime \prime }\left(\omega \right)=\frac{G_{0}i\omega \tau_{c}}{1+i\omega \tau_{c}}$$

yielding:

$$G^{\prime }(\omega )=\frac{G_{0}(\omega \tau_{c}{)}^{2}}{1+(\omega \tau_{c}{)}^{2}},\;G^{\prime \prime }(\omega )=\frac{G_{0}\omega \tau_{c}}{1+(\omega \tau_{c}{)}^{2}}$$

These relations predict a characteristic crossover at $\omega_{c}=1/\tau_{c}$, separating viscous-dominated and elastic-dominated regimes.

However, real hydrogels rarely conform to single-mode Maxwell behavior due to the presence of multiple relaxation mechanisms spanning a broad range of time scales. These include polymer chain dynamics, entanglement effects, reversible crosslinking, and network heterogeneity [20,21,22,23]. As a result, the stress relaxation behavior is more accurately described by a continuous relaxation spectrum H(τ) [24], which decomposes the macroscopic response into a superposition of Maxwell modes:

$$G(t)=\int_{0}^{\infty }H(\tau )exp(-t/\tau )\;dln\;\tau$$

The shape of H(τ) provides direct insight into the hierarchical dynamics of the network. For example, bimodal or multimodal spectra can arise from distinct dynamic bonds with different lifetimes [25], enabling independent tuning of multiple energy dissipation pathways (Figure 2e,f).

When the relaxation spectrum becomes sufficiently broad, the material often exhibits non-exponential, power-law-like relaxation behavior. In such cases, fractional viscoelastic models [29] provide a compact and physically meaningful description by introducing a “spring-pot” element with the constitutive relation:

$$\sigma \left(t\right)=V\frac{d^{\alpha }\gamma \left(t\right)}{dt^{\alpha }}$$

where 0<α<1 and V is a quasi-property with units of $\mathrm{Pa}{\cdot s}^{\alpha }$ [30]. This formulation effectively captures the intermediate behavior between purely elastic and purely viscous responses and is particularly useful for describing critical gels and fractal-like network structures.

Over the past decades, theoretical developments have extended the classical Maxwell framework by explicitly incorporating the role of network microstructure in governing relaxation dynamics [31,32,33,34]. Among these advances, the most influential contributions were made by Leibler, Semenov, Rubinstein, and Colby [32,35,36,37], who developed the so-called sticky Rouse and sticky reptation models for non-entangled and entangled polymer systems, respectively. By introducing reversible associative interactions into classical Rouse and reptation theories and employing scaling analysis, these models provide a molecular-level description of viscoelastic relaxation in transient networks [38]. In particular, they reveal that the overall network relaxation time is primarily governed by the lifetime of individual dynamic bonds, but can be significantly extended by increasing polymer concentration due to changes in chain connectivity and topology.

The applicability of different viscoelastic models is closely related to the distribution of relaxation timescales in hydrogel networks. For systems characterized by a narrow relaxation spectrum with a single dominant timescale, the classical Maxwell model provides a reasonable approximation. However, most hydrogels exhibit multiple relaxation processes arising from heterogeneous crosslinking, chain dynamics, and network topology, for which generalized Maxwell models or continuous relaxation spectra H(τ) offer a more accurate description.

When the relaxation spectrum becomes sufficiently broad and exhibits scale-free characteristics, fractional viscoelastic models provide a compact representation of power-law relaxation behavior. In such systems, the fractional order (0 < α < 1) reflects the degree of structural heterogeneity and multiscale dynamics within the network.

Overall, the selection of an appropriate model is governed by the underlying molecular interactions and their associated timescales, including bond lifetime, network connectivity, and structural hierarchy.

It is important to distinguish between viscoelastic fluid-like polymer systems and percolated hydrogel networks. Classical polymer gels are characterized by a continuous network structure that gives rise to a dominant elastic response over a broad frequency range, typically with the storage modulus G′ exceeding the loss modulus G″. In contrast, viscoelastic fluids exhibit terminal flow behavior at long timescales, where G″ > G′ and stress relaxation leads to complete dissipation of stored energy.

However, many modern hydrogels incorporate dynamic or reversible crosslinking interactions, which introduce time-dependent behavior that can bridge solid-like and fluid-like responses. As a result, the observed mechanical properties depend strongly on the experimental timescale and frequency window. In this context, simplified viscoelastic models such as the Maxwell model may still be used to describe individual relaxation processes or limiting cases, although they do not fully capture the behavior of percolated gel networks [39].

Building upon these developments, recent theoretical frameworks have further emphasized the intrinsic coupling between molecular interactions and network architecture in determining viscoelastic behavior. Rather than being solely controlled by bond lifetimes, relaxation dynamics emerge from the interplay between reversible bonding, chain connectivity, and topological constraints. This perspective highlights the necessity of integrating molecular-scale kinetics with network-scale structure, thereby providing a more unified foundation for understanding viscoelasticity and guiding the rational design of hydrogels with tailored mechanical properties.

## 3. Construction of Hydrogels with Dynamic Mechanical Properties

Dynamic intermolecular and covalent interactions play a central role in determining the mechanical adaptability of hydrogel networks, thereby providing a molecular foundation for regulating viscoelastic behavior across multiple time scales. Depending on the nature and exchange kinetics of these interactions, hydrogels can exhibit markedly different balances between structural stability and stress relaxation. The current viscoelastic hydrogels are primarily constructed based on the following classes of reversible crosslinking mechanisms: (i) ionic interactions characterized by rapid association-dissociation dynamics [40,41,42]; (ii) hydrogen-bond networks with fast but relatively weak reversible connections [43,44,45,46,47]; (iii) hydrophobic associations that provide sustained energy dissipation through transient domains [48,49,50,51]; and (iv) dynamic covalent linkages capable of maintaining long-term structural integrity while allowing controlled bond exchange [52,53,54,55]. These interaction types collectively represent a continuum spanning from rapidly exchanging physical junctions to more stable yet reconfigurable covalent networks. In this section, we summarize their fundamental bonding mechanisms, viscoelastic characteristics, and representative material design strategies.

### 3.1. Ionically Crosslinked Hydrogels

Ionically crosslinked hydrogels are formed through electrostatic interactions or metal-ligand coordination between charged polymer chains and multivalent ions. These ionic bonds are reversible and typically exhibit intermediate binding strength and relatively fast exchange kinetics, allowing the network structure to reorganize under mechanical loading. Such dynamic bond dissociation and reformation provide an intrinsic molecular basis for viscoelastic behavior.

The viscoelasticity of ionically crosslinked hydrogels is primarily governed by the lifetime and density of ionic interactions. Under deformation, partial rupture of ionic crosslinks enables stress redistribution and energy dissipation, while subsequent bond reformation restores network connectivity. Consequently, parameters such as ion valence, coordination geometry, ionic strength, and polymer charge density directly influence stress relaxation, hysteresis, and resilience. Compared with permanent covalent networks, ionic networks generally exhibit faster stress relaxation and enhanced self-recovery, making them suitable for applications involving repeated deformation or dynamic transport processes.

**Figure 3:**
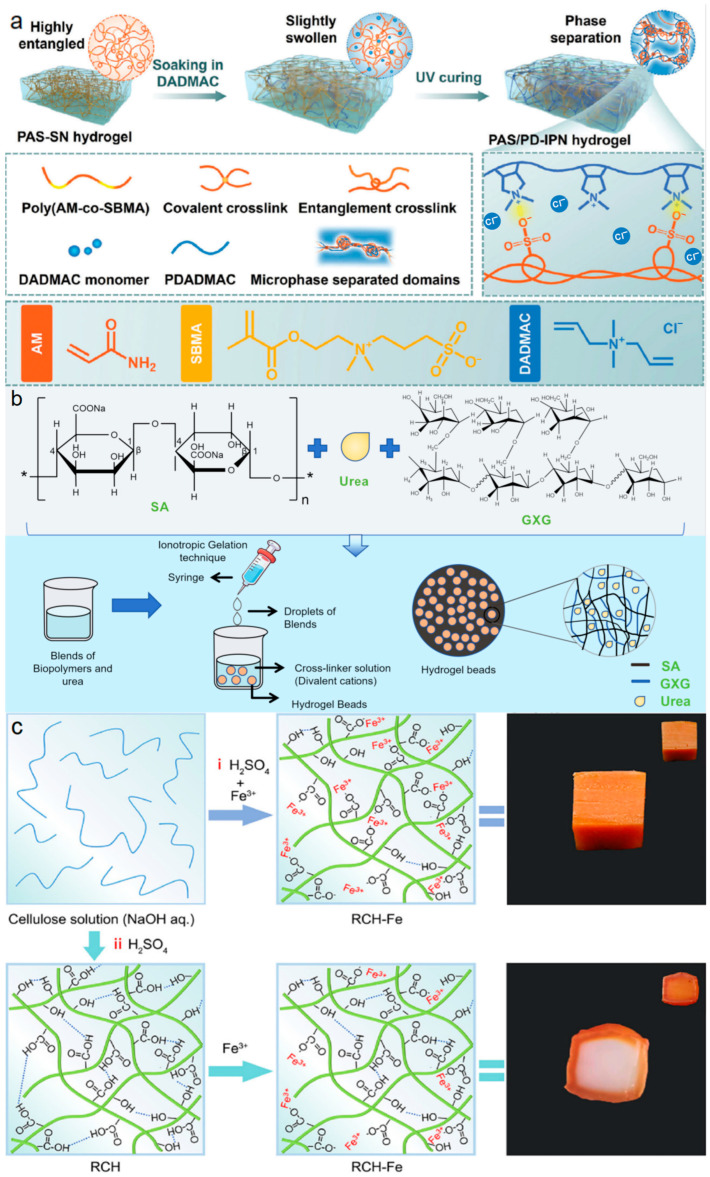
Regulation of viscoelasticity in ionically crosslinked hydrogels. (**a**) Schematic illustration of the design and fabrication of Poly(diallyldimethylammonium chloride) (PAS/PD) interpenetrating polymer network (IPN) hydrogels. Copyright 2024, John Wiley and Sons [56]. (**b**) Schematic representation of the preparation of sodium alginate-Galactoxyloglucan (SA-GXG) ionically crosslinked hydrogels, reproduced based on Balakrishnan M et al., 2024 [57]. (**c**) Representative fabrication strategies of Fe^3+^-cross-linked regenerated cellulose (RCH-Fe) hydrogels: (i) one-step synthesis and (ii) two-step synthesis routes. Insets show the as-prepared hydrogels, and corresponding cross-sectional images highlight differences in internal network structures. Copyright 2026, American Chemical Society [58].

These characteristics have been widely exploited in soft electronic materials, structural carriers, and responsive delivery systems. For instance, the formation of ionic domains through zwitterionic interactions or metal coordination can generate heterogeneous network structures that function as reversible dissipation centers, enabling stable mechanical performance under cyclic loading. Liang and co-workers reported a polyzwitterion/polyelectrolyte interpenetrating network hydrogel in which microphase separation was introduced through zwitterionic units (Figure 3a) [56]. The resulting ionic domains acted as dynamic physical crosslinks that could reversibly reorganize during deformation, leading to high resilience, low mechanical hysteresis, and strain-stiffening behavior. Such viscoelastic characteristics are critical for maintaining signal stability and fatigue resistance in ionic skin devices subjected to repeated mechanical cycling.

Ionically crosslinked networks are also frequently used to regulate mass transport through controlled swelling and structural stabilization. Calcium-ion-mediated crosslinking of alginate is a well-established example, where divalent Ca^2+^ ions coordinate with carboxylate groups to form a three-dimensional network. The reversible nature of this coordination interaction allows the hydrogel to maintain mechanical integrity while undergoing hydration and swelling. Balakrishnan et al. utilized this mechanism to prepare sodium alginate-galactoxyloglucan composite hydrogel beads for slow-release fertilizer applications (Figure 3b) [57]. In this system, the elastic restoring force generated by ionic crosslinks balanced osmotic swelling pressure, enabling sustained water retention and regulated nutrient diffusion. The study illustrates how ionic bond dynamics can simultaneously control mechanical stability and transport behavior in hydrogel carriers.

Metal-ligand coordination provides another effective strategy for enhancing viscoelastic dissipation in mechanically robust hydrogels. Multivalent metal ions, such as Fe^3+^, can form coordination bonds with hydroxyl or carboxyl groups on polymer chains, creating reversible sacrificial bonds within the network. Liu and co-workers prepared Fe^3+^-crosslinked regenerated cellulose hydrogels through a one-step regeneration-crosslinking process in an acidic coagulation bath (Figure 3c) [58]. The homogeneous distribution of Fe^3+^-carboxyl coordination bonds enabled efficient energy dissipation during deformation, as indicated by pronounced hysteresis loops in tensile and compressive tests. This viscoelastic dissipation mechanism allowed the hydrogel to withstand secondary mechanical processing, such as cutting or shaping, while maintaining structural integrity.

Overall, ionically crosslinked hydrogels offer a simple and effective route for regulating viscoelastic properties through dynamic electrostatic interactions and coordination chemistry. Their main advantages include facile fabrication, reversible energy dissipation, and good biocompatibility under aqueous conditions. However, the mechanical stability of ionic networks can be sensitive to environmental factors such as pH, ionic strength, and temperature, and excessive bond mobility may lead to creep or long-term deformation under sustained loading. Future developments are likely to focus on integrating ionic interactions with other dynamic bonding mechanisms to achieve improved mechanical robustness and broader operational stability.

### 3.2. Hydrogen-Bonded Hydrogels

Hydrogen-bonded hydrogels are constructed through reversible intermolecular interactions between functional groups such as hydroxyl, amide, and carboxyl moieties. Compared with ionic or covalent bonds, hydrogen bonds typically possess lower binding energies and shorter lifetimes, enabling rapid bond exchange and network rearrangement under mechanical loading. This dynamic and reversible nature provides an effective molecular mechanism for regulating viscoelastic behavior.

The viscoelasticity of hydrogen-bonded hydrogels is primarily governed by the density and strength of hydrogen-bond interactions as well as their cooperative organization within the network. During deformation, hydrogen bonds can reversibly dissociate to dissipate mechanical energy, while subsequent reformation restores network integrity. This continuous bond rupture-reformation process allows the material to maintain mechanical stability while accommodating large deformations. As a result, hydrogen-bonded hydrogels typically exhibit rapid stress relaxation, efficient energy dissipation, and intrinsic self-healing capability, making them particularly suitable for applications requiring mechanical adaptability in dynamic environments, such as wound dressings, flexible electronics, and wearable sensors.

**Figure 4:**
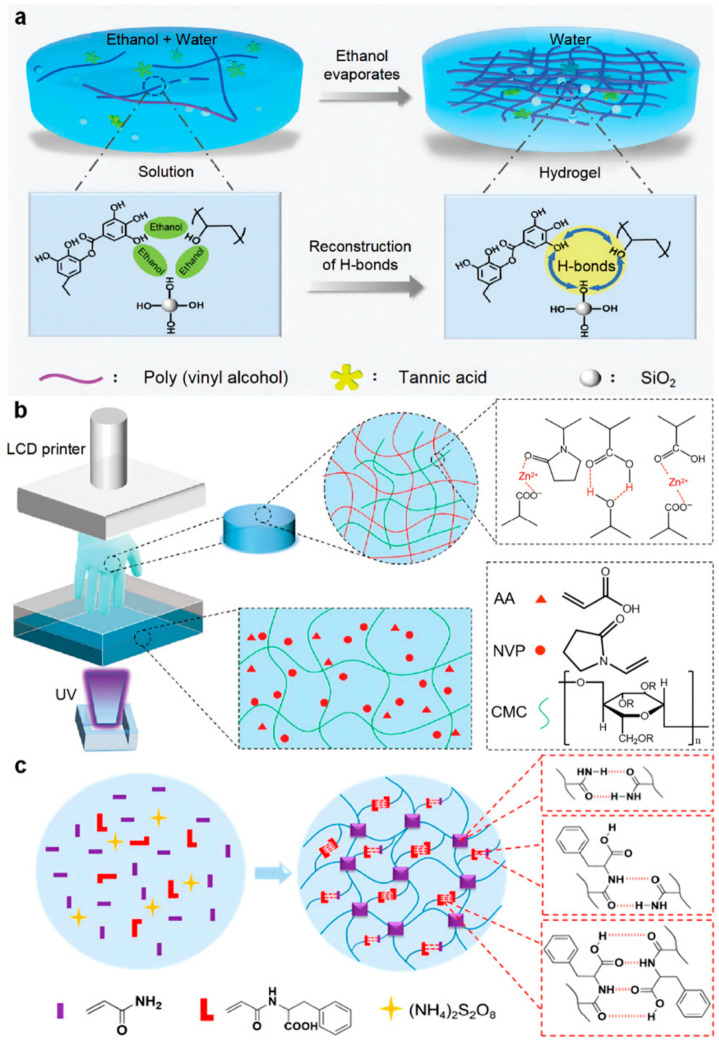
Regulation of viscoelasticity in hydrogen-bonded hydrogels. (**a**) Schematic illustration of reconstruction of hydrogen bond of PVA and TA when the ethanol is evaporated. Copyright 2021, John Wiley and Sons [59]. (**b**) liquid crystal display (LCD) printing of interpenetrated network hydrogels based on physical interactions (H-bonds and Zn^2+^-ligand coordination). Copyright 2021, John Wiley and Sons [60]. (**c**) Dynamic crosslinking assembly. Copyright 2021, American Chemical Society [61].

In the field of harsh-environment oil–water separation, Bai et al., developed a polyvinyl alcohol (PVA) tannic acid (TA) “hydrogel coating” based on solvent (ethanol/water) dynamic regulation of hydrogen bonding (Figure 4a) [59]. The core of this design lies in utilizing ethanol to temporarily shield the strong hydrogen bonds between PVA and TA, maintaining the mixed solution in a homogeneous, coatable fluid state (the “coating” state). Upon coating, as the ethanol evaporates, the hydrogen bonds between PVA and TA rapidly reconstruct to form a dense physical crosslinking network. This dynamic process endows the coating with high strength (>10 MPa) and excellent anti-swelling stability, enabling it to maintain stable mechanical and superwetting properties in acidic and saline environments.

In the field of high-precision customized manufacturing Wu Yuchao et al. developed an interpenetrated network hydrogel using carboxymethyl cellulose (CMC) as the first network, and poly(acrylic acid) (AA) and N-vinyl-2-pyrrolidone (NVP) as the second network interpenetrating network hydrogel suitable for photocurable 3D printing (Figure 4b) [60]. This design constructs a dual physical crosslinking network via Zn^2+^-coordination bonds and hydrogen bonds, replacing traditional chemical crosslinking. Crucially, the physical crosslinking network provides rapid reversible breakage and recombination capabilities, achieving the essential “rapid solid–liquid separation” required during the printing process. The resulting hydrogel exhibits high toughness (3.38 MJ·m^−3^) and efficient self-healing ability; its physical crosslinking network reversibly breaks and reforms during cyclic deformation, ensuring the sensing stability of printed devices.

In the development of ultra-stretchable, fatigue-resistant elastomers Yu Jing et al. prepared hydrogels based on multiple hydrogen bonds via the copolymerization of acrylamide and N-acryloyl phenylalanine (APA) (Figure 4c) [61]. The core of this design lies in utilizing the hydrophobic benzene rings of APA to stabilize multiple hydrogen bonds, forming robust physical crosslinking points within the hydrophilic network. This endows the hydrogel with an exceptional combination of viscoelastic properties: ultra-high stretchability (>2100%), high toughness (tear energy 1134 J·m^−2^), near-perfect elastic recovery, and fatigue resistance, with no performance degradation observed over 100 compression cycles.

Overall, hydrogen-bonded hydrogels provide a versatile platform for regulating viscoelastic properties through reversible and rapidly exchangeable intermolecular interactions. Their major advantages include fast self-healing, efficient energy dissipation, and compatibility with aqueous and biological environments. However, the relatively weak strength of individual hydrogen bonds can limit mechanical stability under high loads or elevated temperatures, and excessive water content may further reduce interaction strength. Future research is expected to focus on hierarchical hydrogen-bond architectures and cooperative multi-interaction networks to achieve improved mechanical robustness while preserving dynamic adaptability.

### 3.3. Hydrophobically Crosslinked Hydrogels

Hydrophobic association introduces transient physical junctions within polymer networks, typically manifested as micelles or hydrophobic domains dispersed in aqueous environments. These dynamic aggregates act as reversible crosslinking nodes that stabilize the network while retaining structural mobility. Compared with hydrogen bonds or ionic interactions, hydrophobic associations generally exhibit moderate stability and slower exchange kinetics, allowing them to sustain repeated deformation while continuously dissipating mechanical energy.

Time-dependent mechanical behavior in hydrophobically associated hydrogels is closely associated with the stability and rearrangement dynamics of hydrophobic domains. Under mechanical loading, partial dissociation or reorganization of these domains permits polymer chain sliding and stress redistribution, thereby preventing catastrophic fracture. Once the external force is removed, hydrophobic segments reassociate spontaneously, restoring network connectivity and mechanical integrity. Parameters such as hydrophobic group content, micelle size distribution, and polymer architecture therefore play decisive roles in determining stress relaxation rate, toughness, and self-healing efficiency. This reversible association-reassociation process provides a reliable molecular basis for achieving durable viscoelastic performance under cyclic deformation.

**Figure 5:**
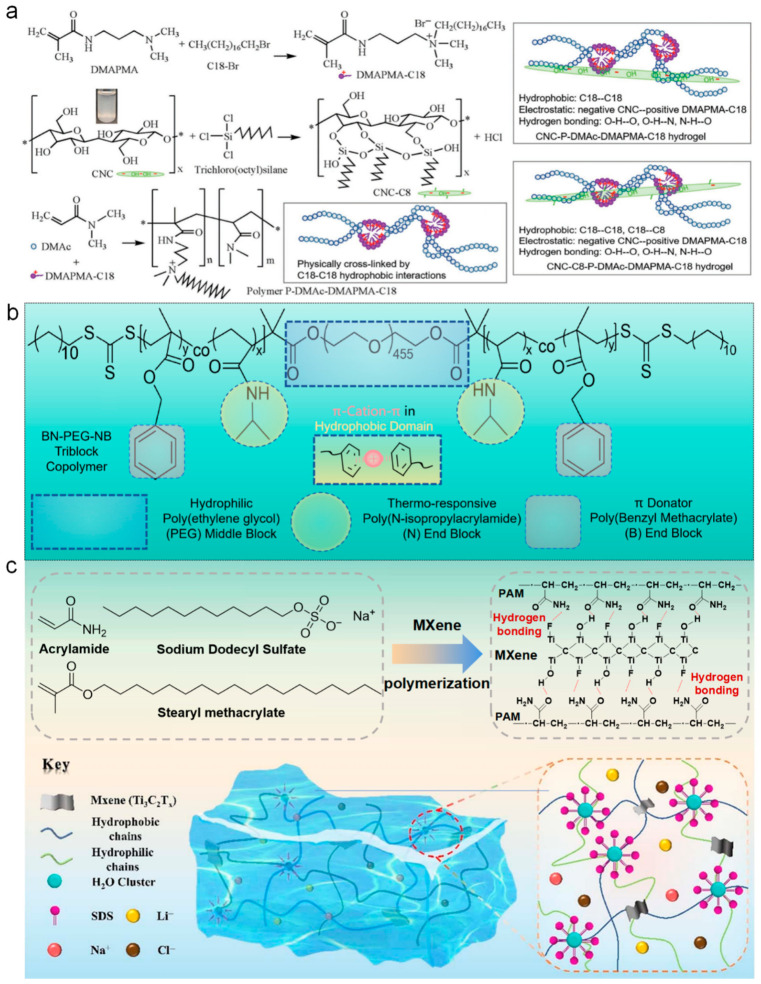
Regulation of viscoelasticity in hydrophobic association hydrogels. (**a**) Schematic illustration of the synthesis of key components, including grafting of octadecyl chains onto N-[3-(dimethylamino)propyl]methacrylamide (DMAPAMA-C18), hydrophobically modified cellulose nanocrystals (CNC-C8) (inset: CNC suspension in toluene), and polymerization of N,N-dimethylacrylamide with DMAPMA-C18 monomers initiated by potassium persulfate (P-DMAc-DMAPMA-C18), as well as the corresponding interaction mechanisms in CNC-based composite hydrogels. Copyright 2020, John Wiley and Sons [62]. (**b**) Chemical structure of an ABA triblock copolymer designed for universal hydrogel wound dressings, in which the three-dimensional network is formed through copolymer assembly driven by synergistic π-cation-π and hydrophobic interactions. This schematic was redrawn based on Xiang L et al., 2026 [63]. (**c**) Schematic representation of the hydrogel fabrication process, with an enlarged view highlighting multiple physical interactions within the network, including hydrophobic association, hydrogen bonding, and MXene-mediated electrostatic crosslinking. Copyright 2013-Present, Royal Society of Chemistry [64].

Combining hydrophobic aggregation with secondary reinforcing interactions represents a practical route toward mechanically robust yet recoverable networks. In amphiphilic polymer systems, hydrophobic microdomains often function as primary energy dissipation units, while rigid fillers or secondary interactions restrict excessive chain mobility. Liu et al. constructed a double physically crosslinked hydrogel reinforced by cellulose nanocrystals (CNC), where hydrophobic associations served as the main reversible junctions and CNC particles introduced additional physical interactions through hydrogen bonding and electrostatic attraction (Figure 5a) [62]. Mechanical loading triggered progressive disruption of hydrophobic domains and interfacial interactions, enabling efficient energy dissipation across multiple length scales. Following damage, reassociation of hydrophobic segments allowed the network to recover its structural integrity, supporting repeated use and convenient repair through solvent treatment.

Hydrophobically associated networks are particularly valuable in environments where mechanical stability must be maintained under variable chemical conditions. Thermoresponsive amphiphilic block copolymers provide a representative platform in which temperature-induced aggregation of hydrophobic segments generates injectable gels capable of rapid network reconstruction. Xiang et al. developed a copolymerizing salt cation-responsive π donator component of benzyl methacrylate (BM) and thermal-responsive N-isopropylacrylamide (NIPAM) as terminal blocks onto both flanks of the middle hydrophilic poly(ethylene glycol) (PEG) block, denoted as poly(BN-PEG-NB) to establish a dynamic viscoelastic network (Figure 5b) [63]. Reversible formation and dissociation of hydrophobic domains enabled rapid modulus recovery after mechanical disruption, even under extreme pH and high-salt conditions simulating gastrointestinal environments. Such adaptive mechanical behavior allows the material to withstand periodic peristaltic motion while maintaining continuous structural protection at the wound interface.

Hydrophobic microdomains can also be integrated with nanostructured components to stabilize mechanical response in flexible electronic systems. In micelle-assisted polymerization processes, hydrophobic monomers are confined within micellar cores that subsequently act as reversible physical junctions in the final network. Xiang Di et al. designed a dual physically crosslinked hydrogel by polymerizing stearyl methacrylate (SMA) within micelles and incorporating two-dimensional MXene nanosheets as reinforcing elements (Figure 5c) [64]. During deformation, disruption of hydrophobic microdomains dissipated mechanical energy, whereas interfacial interactions between MXene surfaces and polymer chains limited excessive chain slippage and promoted rapid recovery of network elasticity. This cooperative mechanism allowed the hydrogel to tolerate repeated large deformations while maintaining stable mechanical and electrical responses, supporting reliable sensing of both subtle and large-amplitude human motion.

Taken together, hydrophobically associated hydrogels provide an effective framework for regulating viscoelastic behavior through reversible aggregation of hydrophobic domains. Their mechanical robustness originates from the capacity of these domains to function as sacrificial units that dissipate energy while preserving network continuity. Nevertheless, the stability of hydrophobic aggregates may depend on temperature, polymer concentration, and solvent conditions, which can introduce variability in mechanical performance across different environments. Continued efforts to control domain size distribution and association kinetics are expected to enable more precise tuning of viscoelastic response over a broad range of time scales.

### 3.4. Dynamic Covalent Hydrogels

Dynamic covalent chemistry introduces reversible covalent linkages capable of undergoing bond exchange under specific chemical or physical conditions. Unlike purely physical interactions, these bonds typically possess higher binding energies while still retaining the ability to reorganize over experimentally relevant time scales. A wide range of dynamic covalent reactions, including thioester exchange [55,65,66], disulfide metathesis [67], hydrazone formation [54,68,69], boronic ester complexation [52,53,70], oxime ligation [71,72,73], Schiff base reactions [74,75,76], and Diels-Alder cycloadditions [77,78,79], has been incorporated into hydrogel networks to achieve controllable mechanical stability combined with time-dependent structural adaptability. This unique balance between stability and reversibility provides an effective molecular framework for tuning viscoelastic response across extended time scales.

The mechanical relaxation behavior of dynamically crosslinked networks is primarily dictated by bond exchange kinetics. In linear viscoelastic regimes, the characteristic relaxation time (τ) reflects the average lifetime of reversible covalent junctions, whereas in nonlinear regimes, the rate of bond dissociation governs strain-dependent energy dissipation and flow behavior. By adjusting parameters such as reaction equilibrium constants, catalyst concentration, and network topology, dynamic covalent systems allow precise modulation of viscoelastic response without compromising structural integrity. This tunability is particularly advantageous in applications where mechanical stability and long-term reliability must coexist with controlled stress relaxation, including wearable electronics, tissue engineering scaffolds, and injectable biomaterials.

**Figure 6:**
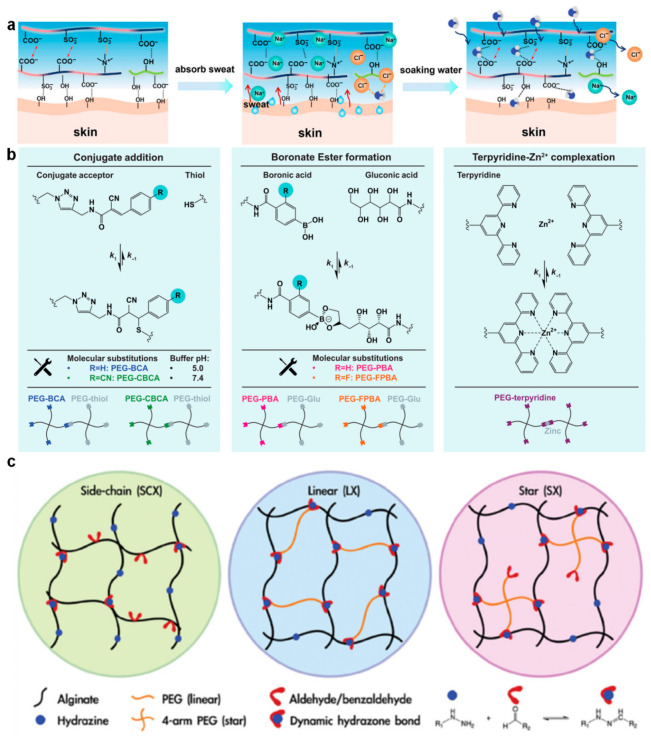
Regulation of viscoelasticity in hydrogels via dynamic covalent interactions. (**a**) Schematic illustration of wet adhesion mechanisms in hydrogels, highlighting the interactions between functional groups and on-demand adhesive chains. Copyright 2026, John Wiley and Sons [80]. (**b**) Representative dynamic crosslinking strategies in 4-armed PEG hydrogels, including conjugate addition, boronate ester formation, and terpyridine-zinc coordination. The bond dissociation kinetics can be tuned through molecular design and environmental conditions, enabling control over network dynamics. Copyright 2026, American Association for the Advancement of Science [81]. (**c**) Schematic representation of dynamically crosslinked (DCC) hydrogels with diverse crosslinker architectures. Copyright 2024, John Wiley and Sons [82].

Environmental responsiveness can further extend the functional range of dynamic covalent hydrogels by coupling bond exchange behavior to external stimuli. For example, ionic strength and solvent composition can influence intermolecular interactions and thereby regulate network density and adhesion performance. Wu et al. developed a poly(acrylic acid-co-sulfobetaine methacrylate-co-laury lmethacrylate)/carboxymethyl cellulose hydrogel (PASLC) hydrogel designed to respond to sodium ions present in human sweat, where electrostatic shielding and salting-out effects modulate the compactness of the network (Figure 6a) [80]. In sweat-containing environments, increased ionic strength stabilizes interchain interactions and maintains strong wet adhesion, whereas exposure to pure water promotes rapid swelling and reduces network cohesion, enabling controlled detachment. The ability to switch between mechanically stable and easily releasable states reflects a reversible adjustment of network structure driven by environmental conditions, providing a practical strategy for balancing adhesion reliability and user comfort in wearable sensing systems.

Systematic studies of dynamic covalent networks have also clarified the quantitative relationship between molecular-scale reaction kinetics and macroscopic viscoelastic behavior. Crowell et al. investigated 4-armed poly(ethylene glycol) (PEG) hydrogels crosslinked through distinct reversible chemistries, including conjugate addition and boronic ester formation, and demonstrated that variation in bond dissociation rate constants directly determines the characteristic relaxation time over several orders of magnitude (Figure 6b) [81]. The onset of nonlinear rheological behavior, such as shear thickening, was found to correlate with a specific Weissenberg number, indicating that macroscopic flow behavior can be predicted from molecular kinetic parameters. These findings provide a clear mechanistic basis for designing hydrogels with predefined viscoelastic time scales by selecting dynamic covalent reactions with appropriate exchange rates.

Network topology represents another critical parameter for regulating viscoelastic performance in dynamic covalent systems. In hydrazone-crosslinked alginate hydrogels, the spatial arrangement of crosslinkers influences both stiffness and relaxation dynamics by altering the distribution of load-bearing junctions. Yung-Hao Lin et al. compared side-chain, linear, and star-shaped crosslinkers and demonstrated that each topology produces a distinct viscoelastic window defined by the relationship between modulus and stress relaxation rate (Figure 6c) [82]. Star-shaped architectures, for example, provide multiple connection points within a confined volume, enabling efficient load transfer while preserving bond exchange capability. This topology-dependent control of viscoelastic response offers practical design guidelines for tailoring hydrogels to match the mechanical requirements of biological microenvironments, such as those encountered in three-dimensional cell culture.

Collectively, dynamic covalent hydrogels provide a versatile platform for regulating viscoelastic behavior through controllable bond exchange kinetics and network architecture. Their defining advantage lies in the ability to maintain structural stability while enabling predictable stress relaxation over extended time scales. Remaining challenges include achieving rapid bond exchange without sacrificing mechanical strength and ensuring long-term stability under physiological conditions. Continued advances in reaction design and network topology are expected to enable increasingly precise control of viscoelastic properties for advanced biomedical and soft electronic applications.

The diverse classes of reversible crosslinking interactions discussed above demonstrate that hydrogel viscoelasticity can be systematically regulated by controlling bond strength, lifetime, and network architecture. Rapidly exchanging interactions enable fast stress relaxation and self-healing, whereas more stable interactions support mechanical robustness and sustained load-bearing capability. Rather than representing isolated design strategies, these mechanisms define an integrated framework for tuning the viscoelastic window of hydrogels to match specific functional requirements. Continued efforts to couple molecular-level interaction design with quantitative rheological characterization will facilitate the development of next-generation hydrogel systems capable of operating reliably in complex and dynamic mechanical environments.

## 4. Microscopic Mechanical Insights from Single-Molecule Force Spectroscopy and the Implications for Hydrogel Design

**Figure 7:**
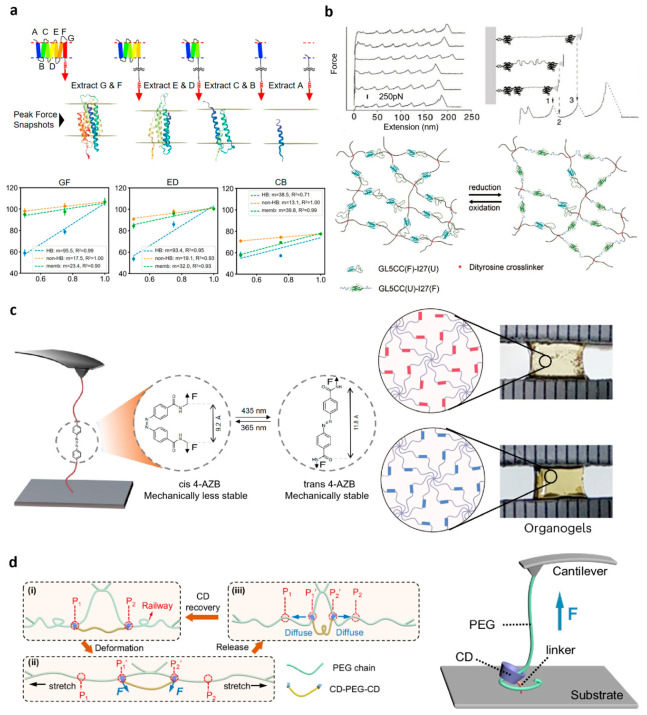
Unraveling Topology and Isomerization Mechanisms via Single-Molecule Force Spectroscopy. (**a**) The protein topology and main stages of pulling, along with snapshots from a replica near a rupture event. Copyright 2023, MDPI [83]. (**b**) Force-extension curves of Ig8 domain stretching (folded/unfolded states) and schematic of redox-responsive GL5CC-I27 MEP protein hydrogels. Copyright 2025, John Wiley and Sons [87]. (**c**) SMFS characterization of 4-AZB isomerization (conformation/mechanical stability) and optical/schematic images of 4-AZB-PEG organogels under 365/435 nm light. Copyright 2023, Springer Nature [90]. (**d**) Schematic of AFM-based SMFS for PEG-CD friction and CD sliding energy dissipation in hydrogels, with chain walker position recovery via diffusion after stress relaxation. Copyright 2024, Springer Nature [94].

Single-molecule force spectroscopy (SMFS) enables direct, quantitative measurement of molecular responses to mechanical force, including folding-unfolding transitions, bond rupture, and non-covalent interaction dynamics (Figure 7a) [83]. These experiments provide key parameters, such as energy barriers, force-dependent kinetics, and mechanically sensitive domains, that reveal molecular stability and tunable mechanical behavior. Such insights are highly relevant for hydrogel design, where macroscopic viscoelasticity emerges from the mechanical properties of network strands and dynamic crosslinks. By integrating SMFS data from proteins or polymers into network models, it becomes possible to predict stress dissipation, relaxation, and self-recovery, establishing a rational, multiscale strategy for designing hydrogels with controllable mechanical properties [84].

The establishment of quantitative correlations between molecular-scale dynamics and macroscopic viscoelastic behavior remains a central challenge in soft matter mechanics. Recent advances have begun to address this gap through complementary approaches that directly probe either network structure or bond-level dynamics. For example, Rosenmann et al. employed liquid-phase transmission electron microscopy (LP-TEM) to visualize micrometer-scale fibrillar assemblies in methylcellulose hydrogels, demonstrating that the high persistence length of bundled nanofibers governs bulk rheological responses, with predictions from modified rheological models in close agreement with experimental data. Complementing this structural perspective, Sanoja and Creton highlighted the role of mechanochemistry in linking molecular events to macroscopic mechanics, showing that the activation of force-sensitive mechanophores at load-bearing sites enables quantitative mapping of stress fields and bond scission processes.

These studies underscore that both network architecture and bond-level dynamics provide critical entry points for quantitatively connecting molecular design to macroscopic viscoelasticity. Building on these advances, it is therefore essential to develop a unified multiscale framework that integrates molecular interactions, network dynamics, and bulk mechanical response. Such a framework forms the basis for the quantitative description of viscoelastic behavior presented below.

To establish a quantitative connection between molecular interactions and macroscopic viscoelasticity, it is necessary to introduce the concept of relaxation timescale distribution. At the molecular level, the dynamics of reversible interactions can be characterized by their bond lifetime, which is inversely related to the dissociation rate constant ($\tau_{b}\approx 1/k_{off}$). These molecular timescales are directly translated into network-level relaxation processes, contributing to the overall relaxation spectrum H(τ).

Within this framework, the macroscopic stress relaxation behavior can be expressed as a superposition of relaxation modes:

$$G(t)=\int_{0}^{\infty }H(\tau )e^{-t/\tau }\;dln\tau$$

where H(τ) reflects the distribution of relaxation processes arising from heterogeneous molecular interactions and network structures.

Different types of molecular interactions contribute to distinct regions of the relaxation spectrum. Short-lived interactions, such as hydrogen bonds, typically dominate fast relaxation processes and enhance viscous dissipation, while longer-lived interactions, such as dynamic covalent bonds, contribute to sustained elasticity at longer timescales. Systems containing multiple interaction types often exhibit broad or multimodal relaxation spectra, enabling decoupled control over elasticity and dissipation.

Importantly, the effective relaxation behavior is not solely determined by bond lifetime, but also depends on network topology, chain connectivity, and polymer concentration, as described by transient network theories such as sticky Rouse and reptation models. This multiscale perspective provides a quantitative foundation for predicting and tuning viscoelastic properties through molecular design.

### 4.1. Protein-Based Single-Molecule Force Spectroscopy for the Design of Viscoelastic Hydrogels

Proteins possess highly programmable mechanical domains, making them ideal building blocks for designing viscoelastic materials. SMFS experiments provide direct insights into the mechanical unfolding/refolding behavior of protein domains, including unfolding forces, energy landscapes, and kinetic rates under varying loading conditions. These molecular characteristics play a decisive role in determining the macroscopic viscoelasticity of protein-based hydrogels.

Within hydrogel networks, mechanically labile protein domains can serve as molecular-scale energy dissipators. Upon mechanical loading, these domains undergo force-induced unfolding, absorbing energy and thereby increasing the loss modulus and enhancing stress relaxation [85]. By tuning the number, sequence arrangement, and mechanical stability of these domains, it is possible to precisely regulate both linear and nonlinear viscoelastic responses. For example, incorporation of mechanically robust immunoglobulin (Ig)-like domains into polymer networks enables substantial energy dissipation under high strain while maintaining structural integrity at low deformation [86].

Notably, the group of Hongbin Li has systematically characterized the unfolding behavior of the GB1 protein using SMFS and further translated these insights into the design of protein-based hydrogels with enhanced fatigue resistance (Figure 7b) [87]. In addition, reversible non-covalent interactions between proteins—such as metal coordination, hydrophobic interactions, and hydrogen bonding—can be quantitatively probed at the single-molecule level, enabling prediction of their dynamic rupture and reformation behavior within macroscopic networks.

Representative systems include hydrogels incorporating titin-derived elastic domains, which exhibit force-induced unfolding and partial refolding, resulting in bimodal mechanical behavior that combines elasticity and dissipation [88]. By modulating the type and abundance of such domains, key parameters such as stress relaxation timescales and cyclic loading stability can be finely tuned. These studies exemplify a molecularly informed design paradigm, in which single-molecule mechanical properties are directly translated into controllable bulk viscoelasticity.

### 4.2. Mechanochemical Single-Molecule Force Spectroscopy for Dynamically Responsive Hydrogel Design

Mechanochemistry focuses on force-induced chemical transformations and conformational changes at the molecular level, and SMFS has emerged as a central tool for probing such processes. These experiments reveal how external forces modulate reaction pathways, including bond rupture, reversible ring-opening reactions, and force-triggered configurational transitions. Such molecular-level insights provide a unique foundation for designing hydrogels with adaptive and stimuli-responsive viscoelastic properties.

In hydrogel systems, mechanochemically active units can function as force-sensitive crosslinks or “mechanical switches”, enabling dynamic energy dissipation, self-healing, and shape adaptation. SMFS allows quantitative determination of key parameters such as rupture forces, force-accelerated reaction rates, and reversibility, which can be directly correlated with macroscopic viscoelastic behavior. For instance, hydrogels incorporating dynamic crosslinks-such as disulfide bonds, hydrophobic assemblies, or metal-ligand coordination-can undergo force-induced bond dissociation and reformation, leading to pronounced nonlinear stress–strain behavior and enhanced energy dissipation [89].

Importantly, force-induced conformational transitions of mechanophores represent another critical design dimension. The group of Jie Li demonstrated that cis and trans para-azobenzene isomers exhibit distinct mechanical properties at the single-molecule level (Figure 7c) [90]. By exploiting light-induced isomerization, they achieved rational control over the macroscopic fracture behavior of polymer networks. This strategy highlights how molecular conformational switching can be harnessed to engineer hydrogels with light-regulated mechanical properties.

Overall, mechanochemical SMFS establishes a direct link between molecular transformations and bulk viscoelasticity, enabling predictive design of hydrogels with programmable mechanical responses across multiple length and time scales.

### 4.3. Slide-Ring Hydrogels: Topological Control of Sliding Dynamics and Relaxation Behavior

Slide-ring hydrogels represent a unique class of topological polymer networks based on mechanically interlocked molecules (MIMs), in which crosslinking junctions are not fixed but can freely slide along polymer chains. This distinctive architecture endows the network with unconventional stress distribution and energy dissipation mechanisms [91].

At the molecular level, the defining feature of slide-ring systems lies in the mobility of ring components along an axial polymer chain. Single-molecule stretching experiments provide direct access to the sliding behavior, enabling measurement of sliding force thresholds, frictional resistance, and associated conformational dynamics. These studies reveal that, unlike conventional covalently crosslinked networks, sliding junctions can dynamically redistribute tension along polymer strands, thereby mitigating stress concentration. This “pulley effect” underlies the exceptional extensibility and fatigue resistance of slide-ring hydrogels [92].

On the materials side, slide-ring hydrogels-typically constructed from polyrotaxanes composed of polyethylene glycol (PEG) threaded through cyclodextrins (CDs)-have been extensively developed since their introduction by Ito Kohzo [93]. By tuning parameters such as cyclodextrin coverage, crosslinking density, and end-capping efficiency, researchers have achieved precise control over mechanical properties ranging from soft elasticity to pronounced viscoelastic dissipation. For example, increasing ring mobility enhances stress relaxation and energy dissipation, whereas restricting sliding leads to more elastic-dominated behavior.

Recent studies further highlight the importance of molecular friction in governing macroscopic viscoelasticity. The group of Xue investigated the molecular friction mechanism of cyclodextrin sliding along PEG chains at the single-molecule level (Figure 7d) [94]. By employing short-chain crosslinked CDs as sliding junctions, they introduced effective chain friction that enables efficient mechanical energy dissipation during deformation, leading to rapid self-recovery and protection of encapsulated components in hydrogel systems.

Moreover, modulation of host–guest interactions through hydrophobic modification or electrostatic interactions provides an additional handle to tune sliding friction and relaxation dynamics [95]. At the application level, slide-ring hydrogels have been widely explored for stretchable electronics, soft robotics, and biomedical scaffolds, owing to their high toughness, low hysteresis, and excellent fatigue resistance [96].

Crucially, SMFS plays a central role in bridging molecular behavior and material performance in these systems. Key single-molecule parameters, such as sliding energy barriers, friction coefficients, and sliding kinetics, can be directly mapped onto macroscopic viscoelastic properties. For instance, lower sliding barriers correspond to faster stress relaxation and higher dissipation, whereas increased friction enhances elastic response and prolongs relaxation timescales [97].

Thus, SMFS-informed design enables rational tuning of slide-ring hydrogels by controlling ring-chain interactions, ring density, and auxiliary non-covalent interactions. This establishes a clear structure-property relationship spanning from molecular topology to bulk mechanics, providing a powerful framework for engineering hydrogels with precisely tailored viscoelasticity.

### 4.4. Quantitative Correlation Between Single-Molecule Mechanical Measurements and Macroscopic Mechanical Properties

A quantitative connection between single-molecule measurements and macroscopic mechanical properties can be established by mapping molecular parameters obtained from single-molecule force spectroscopy (SMFS) onto network-level mechanical descriptors. At the molecular level, the bond lifetime, which can be estimated from force-dependent dissociation kinetics ($\tau_{b}\approx 1/k_{off}$), directly determines the characteristic relaxation timescale of the network and thus governs stress relaxation behavior.

In addition, parameters such as rupture force and activation energy barrier (ΔG^‡^) define the stability of load-bearing interactions and influence nonlinear mechanical responses, including strain-dependent dissipation and fracture. Under applied force, bond dissociation follows force-accelerated kinetics that can be described by Bell–Evans-type relations, linking molecular rupture events to loading-rate-dependent macroscopic behavior. At the network level, the macroscopic modulus is determined by the density of elastically active strands, often approximated as G∼νk_B_T, where ν represents the effective number density of load-bearing chains.

These quantitative relationships provide a general multiscale framework for connecting molecular mechanics to bulk viscoelasticity. For example, Sanoja and Creton demonstrated that embedding mechanophores at load-bearing positions enables quantitative mapping of stress fields and bond scission through optical signals, thereby linking molecular events to macroscopic fracture behavior. Similarly, in protein-engineered hydrogels, Li et al. designed mutually exclusive protein domains whose force-induced unfolding directly correlates with macroscopic energy dissipation under cyclic loading, achieving quantitative agreement between single-molecule mechanics and bulk mechanical response.

## 5. Multiscale Characterization of Viscoelasticity

Accurate evaluation of hydrogel viscoelasticity is essential for understanding energy dissipation, stress relaxation, and dynamic response in both biological and engineering contexts. Characterization spans multiple length scales, from macroscopic rheology to microscale probing, capturing the interplay between network architecture, crosslinking dynamics, and polymer chain mechanics. Macroscopic tests quantify bulk responses under controlled stress or strain, while microscale techniques reveal local heterogeneity and nanoscale viscoelastic behavior. Integrating insights across scales provides a comprehensive framework for correlating molecular- or network-level features with emergent hydrogel mechanics.

### 5.1. Macroscale Viscoelastic Characterization

**Figure 8:**
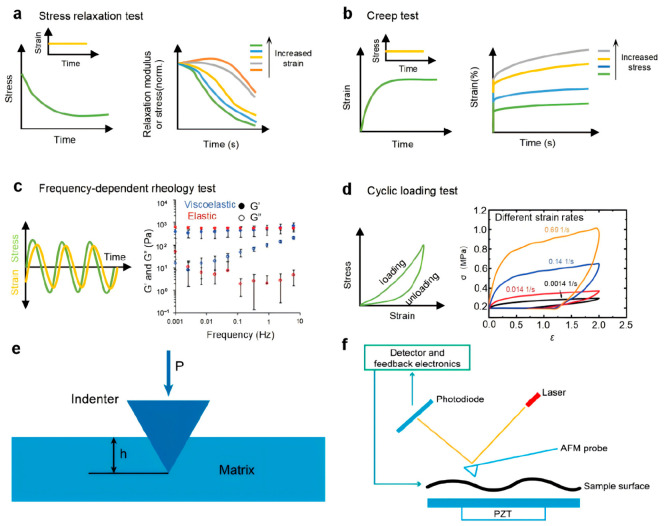
Multiscale characterization of hydrogel viscoelasticity from bulk rheology to microscale mechanics. (**a**) Stress relaxation tests performed under applied constant strain, showing normalized stress relaxation curves of collagen gels at different strain levels. The stress is directly proportional to the relaxation modulus during the test, reproduced based on Nam S et al., 2016 [98]. (**b**) constant stress is applied during the creep test, and creep tests are performed on collagen gels under varying stress levels, reproduced based on Nam S et al., 2016 [98]. (**c**) Frequency-dependent rheological characterization: oscillatory loading with sinusoidal stress or strain, with frequency sweeps yielding storage ($G^{\prime }$) and loss ($G^{\prime \prime }$) moduli that represent elastic and viscous responses, respectively, this schematic was redrawn based on Hui E et al., 2019 [101]. (**d**) Cyclic loading tests: hysteresis in stress–strain curves during loading-unloading cycles, indicating energy dissipation, along with representative cyclic responses at different strain rates, reproduced based on Sun T L et al., 2017 [104]. (**e**) Depth-sensing nanoindentation for probing local mechanical properties. Copyright 2023, MDPI [116]. (**f**) Atomic force microscopy (AFM)-based indentation for nanoscale mechanical characterization. Copyright 2023, MDPI [116].

At the macroscale, hydrogel viscoelasticity is primarily evaluated using static and dynamic mechanical tests. Static methods include stress relaxation and creep measurements. Stress relaxation quantifies the gradual decrease in stress σ(t) under a constant strain $\left(\varepsilon_{0}\right)$, reflecting the network’s ability to redistribute stress over time (Figure 8a). For example, collagen gels display accelerated relaxation at higher strains, indicating that applied deformation facilitates fiber dissociation and energy dissipation within the network. Similarly, creep measures the time-dependent strain response ε(t) under a constant stress σ (Figure 8b). In collagen gels, higher applied stress enhances fiber dissociation, leading to greater creep, demonstrating the nonlinear, stress-dependent nature of viscoelastic response [98]. Characteristic times, such as half-stress relaxation time $\left(\tau_{1/2}\right)$ or creep time $\left(\tau_{3/2}\right)$, provide quantitative metrics for network dynamics and facilitate direct comparison between different hydrogels.

Dynamic mechanical tests complement static measurements by probing time- or frequency-dependent behavior. Frequency-dependent rheology applies oscillatory stress or strain and measures in-phase and out-of-phase responses, corresponding to storage $\left(G^{\prime }\right)$ and loss $\left(G^{\prime \prime }\right)$ moduli (Figure 8c), respectively. Viscoelastic hydrogels typically show frequency-dependent moduli, whereas purely elastic networks remain nearly constant [99,100]. For instance, hyaluronic acid (HA) hydrogels containing both covalent and supramolecular interactions exhibit increasing loss modulus at higher frequencies, reflecting faster disruption and reassociation of host–guest bonds [101]. The loss tangent $\left(tan\;\delta =G^{\prime \prime }/G^{\prime }\right)$ quantifies the viscous contribution and indicates how energy is dissipated under cyclic or dynamic loading. Cyclic loading tests further reveal viscoelastic behavior through hysteresis loops in stress–strain curves (Figure 8d). The loop area directly correlates with energy dissipation, which depends on loading rate and amplitude [102,103]. Gong’s group demonstrated that polyzwitterionic hydrogels exhibit rate-dependent hysteresis growth, as faster cycling promotes the rupture of more ionic bonds, enhancing energy dissipation [104]. Together, these macroscopic methods not only characterize resilience, fatigue resistance, and dynamic load tolerance, but also provide design guidance for tuning crosslink density, chain architecture, and reversible interactions to achieve targeted viscoelastic properties.

### 5.2. Characterization of Viscoelasticity at the Microscale

Microscale techniques probe viscoelasticity at length scales relevant to cellular interactions and local network heterogeneities [105]. Indentation-based methods, such as depth-sensing nanoindentation [106,107,108] and AFM indentation [109,110,111], apply calibrated forces to measure localized mechanical responses. In nanoindentation, a probe applies force perpendicular to the surface, and displacement is recorded, revealing elastic recovery, plastic deformation, and time-dependent stress relaxation (Figure 8e) [112]. This allows calculation of local Young’s modulus and characterization of creep behavior. Frequency-modulated nanoindentation can perform dynamic mechanical analysis (DMA), quantifying $G^{\prime }$ and $G^{\prime \prime }$ at microscale resolution [113]. For example, oligo(ethylene glycol) (OEG) hydrogels exhibit frequency-dependent loss modulus, highlighting microscale network rearrangements not apparent in bulk rheology.

AFM-based indentation combines nanoscale topographic mapping with mechanical probing. A microcantilever with a sharp tip interacts with the sample surface, detecting piconewton-scale forces and generating force-distance curves (Figure 8f). These measurements allow determination of local stiffness, stress relaxation, and creep, providing high-resolution viscoelastic maps. AFM has been applied to alginate hydrogels to evaluate local Young’s modulus [114] and to perform microscale stress relaxation tests on chondrosarcoma cells, revealing intrinsic viscoelastic characteristics at the cellular level [115]. Oscillatory AFM indentation further enables dynamic evaluation of local $G^{\prime }$ and $G^{\prime \prime }$, connecting nanoscale bond dynamics or polymer entanglements to macroscopic energy dissipation. By correlating microscale heterogeneity with bulk mechanical properties, these techniques inform rational hydrogel design, highlighting how local crosslink density, fiber alignment, or dynamic bond kinetics influence overall viscoelastic performance and functional outcomes in biomedical and mechanosensitive applications.

From an experimental perspective, the extraction of the continuous relaxation spectrum H(τ) from rheological data is an inherently ill-posed inverse problem. In practice, H(τ) can be estimated either by fitting stress relaxation data G(t) using a discrete generalized Maxwell model (Prony series), or by reconstructing the spectrum from oscillatory rheology data ($G^{\prime }(\omega )$, $G^{\prime \prime }(\omega )$) through numerical inversion procedures.

To obtain stable and physically meaningful solutions, regularization methods are typically required. For example, Tikhonov regularization has been widely applied to reconstruct relaxation spectra in dynamic polymer networks, enabling reliable estimation even in the presence of experimental noise and limited measurement windows. However, the resolution of the recovered spectrum is inherently limited by factors such as overlapping relaxation modes, incomplete relaxation processes, and finite experimental time ranges, which may obscure fine structural features in the relaxation spectrum [117].

Multiscale characterization of hydrogel viscoelasticity integrates macroscopic mechanical testing with microscale probing to reveal both bulk and local mechanical behavior. Macroscale methods quantify energy dissipation, stress relaxation, and hysteresis, essential for predicting fatigue resistance and dynamic performance. Microscale techniques uncover heterogeneities and local viscoelastic responses that dictate cell–matrix interactions or localized energy dissipation. Together, these approaches provide a framework for correlating molecular- and network-level design with emergent material properties. Future advances may leverage high-resolution, real-time mapping and machine-learning-assisted analysis to enable predictive, multiscale design of hydrogels with precisely tunable viscoelasticity across biomedical, wearable, and soft robotic applications.

## 6. Applications and Outlook

Hydrogels with well-defined viscoelastic properties have demonstrated significant potential in biomedical applications, particularly in tissue regeneration where the mechanical microenvironment plays a decisive role in guiding cellular behavior. Native tissues such as skin, blood vessels, and periodontal ligaments inherently exhibit viscoelasticity, and their function relies critically on time-dependent mechanical responses. Accordingly, engineering hydrogels with tissue-matched viscoelasticity, especially stress relaxation characteristics, has emerged as an effective strategy to promote cell proliferation, differentiation, and extracellular matrix remodeling. Rather than solely matching static stiffness, recent studies highlight that the temporal aspects of mechanical cues can independently regulate biological processes, offering new opportunities for designing biomimetic materials.

In particular, stress-relaxation-controlled hydrogels have been shown to enhance tissue regeneration outcomes by facilitating cell spreading, matrix deposition, and mechanotransduction. For example, hydrogels with relaxation behaviors comparable to native skin can improve collagen deposition and epidermal maturation, while those mimicking the viscoelastic properties of periodontal tissues can regulate stem cell differentiation and promote tissue repair [118,119]. These findings collectively underscore that viscoelasticity is not merely a passive material property, but an active regulatory parameter that governs cell–matrix interactions across multiple scales.

Despite these advances, several fundamental challenges remain. A major limitation lies in the incomplete understanding of how viscoelasticity interacts with biochemical signaling pathways to co-regulate cellular responses. In addition, current theoretical models are still insufficient to describe nonlinear viscoelastic behaviors, such as strain hardening, damage evolution, and force-induced network reorganization under large deformations. Furthermore, the dynamic responses of cells to time-dependent mechanical environments in three-dimensional systems remain poorly understood, particularly with respect to long-term adaptation and feedback mechanisms.

Future research should focus on developing integrated design strategies that combine dynamic covalent chemistry and supramolecular interactions to achieve spatiotemporally programmable viscoelasticity. Advances in multiscale characterization techniques, together with data-driven modeling and simulation, will be essential for establishing quantitative relationships between molecular structure, network dynamics, and macroscopic mechanical properties. Moreover, a deeper understanding of tissue-specific mechanobiological responses, including stem cell fate regulation and epigenetic effects, will further enable the rational design of next-generation hydrogels. With continued progress in these areas, hydrogels with programmable viscoelasticity are expected to play an increasingly important role in tissue engineering, organoid development, and advanced biomedical applications.

## 7. Conclusions

In this review, we have presented a comprehensive perspective on the viscoelastic behavior of hydrogels, spanning from fundamental theoretical frameworks to molecular-level insights and macroscopic characterization. We first outlined the core principles governing viscoelasticity in polymer networks, emphasizing the roles of relaxation spectra, dynamic bonding, and network topology. We then discussed recent advances in the design of hydrogels with dynamic mechanical properties, highlighting how reversible interactions and adaptive network architectures enable tunable energy dissipation and time-dependent mechanical responses. Furthermore, we summarized emerging insights from single-molecule force spectroscopy, which provide direct access to bond-level mechanics and kinetics, offering a powerful bridge between molecular interactions and bulk mechanical behavior. Finally, we reviewed multiscale experimental approaches that enable the characterization of viscoelasticity across different length and time scales, facilitating a more integrated understanding of hydrogel mechanics.

Despite significant progress, several fundamental challenges remain. In particular, establishing quantitative and predictive relationships between molecular-scale dynamics and macroscopic viscoelastic properties remains an open problem. Future research should focus on developing unified multiscale frameworks that integrate molecular mechanics, network dynamics, and continuum descriptions, as well as on advancing in situ and time-resolved characterization techniques. In addition, the incorporation of force-responsive chemistries and programmable dynamic interactions offers new opportunities for designing hydrogels with adaptive and self-regulating mechanical properties.

A deeper understanding of hydrogel viscoelasticity is not only critical for advancing soft materials science, but also has far-reaching implications across disciplines, including biomechanics, tissue engineering, soft robotics, and bioinspired materials. By bridging molecular mechanisms with macroscopic performance, studies of viscoelastic hydrogels are expected to enable the rational design of next-generation materials that more closely mimic the dynamic and adaptive nature of biological systems.

## Data Availability

No new data were created or analyzed in this study. Data sharing is not applicable to this article.
